# M3814, a DNA-PK Inhibitor, Modulates ABCG2-Mediated Multidrug Resistance in Lung Cancer Cells

**DOI:** 10.3389/fonc.2020.00674

**Published:** 2020-05-12

**Authors:** Zhuo-Xun Wu, Zheng Peng, Yuqi Yang, Jing-Quan Wang, Qiu-Xu Teng, Zi-Ning Lei, Yi-Ge Fu, Ketankumar Patel, Lili Liu, Lizhu Lin, Chang Zou, Zhe-Sheng Chen

**Affiliations:** ^1^Department of Pharmaceutical Sciences, College of Pharmacy and Health Sciences, St. John's University, Queens, NY, United States; ^2^The Second Clinical Medical College of Jinan University, The First Affiliated Hospital of Southern University of Science and Technology, Shenzhen People's Hospital, Shenzhen, China; ^3^Guangdong Provincial Key Laboratory of Occupational Disease Prevention and Treatment, Guangdong Province Hospital for Occupational Disease Prevention and Treatment, Guangzhou, China; ^4^Cancer Center, The First Affiliated Hospital of Guangzhou University of Chinese Medicine, Guangzhou, China

**Keywords:** ATP-binding cassette (ABC) transporter, M3814, nedisertib, ABCG2, multidrug resistance (MDR)

## Abstract

M3814, also known as nedisertib, is a potent and selective DNA-dependent protein kinase (DNA-PK) inhibitor under phase 2 clinical trials. ABCG2 is a member of the ATP-binding cassette (ABC) transporter family that is closely related to multidrug resistance (MDR) in cancer treatment. In this study, we demonstrated that M3814 can modulate the function of ABCG2 and overcome ABCG2-mediated MDR. Mechanistic studies showed that M3814 can attenuate the efflux activity of ABCG2 transporter, leading to increased ABCG2 substrate drugs accumulation. Furthermore, M3814 can stimulate the ABCG2 ATPase activity in a concentration-dependent manner without affecting the ABCG2 protein expression or cell surface localization of ABCG2. Moreover, the molecular docking analysis indicated a high affinity between M3814 and ABCG2 transporter at the drug-binding cavity. Taken together, our work reveals M3814 as an ABCG2 modulator and provides a potential combination of co-administering M3814 with ABCG2 substrate-drugs to overcome MDR.

## Introduction

Lung cancer remains the leading cause of cancer-related mortality worldwide (1). Approximately 85% of the cases are characterized as non-small cell lung cancer (NSCLC) (2). Currently, the clinical treatment strategies include surgery, radiotherapy, and chemotherapy (3). Adjuvant chemotherapy including cisplatin, paclitaxel, docetaxel, gemcitabine, and irinotecan, has been accepted as standard treatment especially for patients with advanced NSCLC (4, 5). Another promising option is the usage of small-molecule inhibitors such as gefitinib and erlotinib (6). However, the high rate of metastasis and drug resistance maintains the continued high mortality rates of lung cancer.

Chemotherapy and radiotherapy can lead to DNA damage, the mechanism by which some anticancer drugs, such as etoposide and doxorubicin, exert their anticancer effect (7). DNA-dependent protein kinase (DNA-PK) is actively involved in the repair of DNA double-strand breaks (DSBs), thereby positioning it as a promising target for cancer treatment (8). Several potent DNA-PK inhibitors have been developed, such as VX-984, NU7427, and M3814 (nedisertib) (9, 10). Among all, M3814 is a clinical-stage, highly potent and selective DNA-PK inhibitor that demonstrated high activity in preclinical models (11, 12). M3814 has shown promising activity in combination with etoposide and cisplatin in lung cancer xenograft models (7, 13). It is also being investigated as monotherapy for solid tumors and chronic lymphocytic leukemia (CLL, ClinicalTrials.gov ID: NCT02316197), and as part of a combination treatment with radio-chemotherapy (NCT02516813, NCT03770689).

ATP-binding cassette (ABC) transporter family is composed of membrane proteins that serve multiple biological functions. ABC transporters are widely expressed in different organs such as the blood-brain barrier (BBB), placenta, and small intestines, to protect the organs by extrusion of xenobiotics and toxins. However, several ABC transporters are associated with multidrug resistance (MDR) and confer resistance to multiple chemotherapeutic agents as well as some tyrosine kinase inhibitors (TKIs) (14–16). Studies have shown that ABCB1 (P-glycoprotein, MDR1) and ABCG2 (BCRP, MXR) are associated with drug resistance in the clinical setting (17–19). ABCG2 overexpression can render cancer cells resistant to conventional chemotherapeutic agents, in particular topotecan, irinotecan, mitoxantrone, and doxorubicin (20–22), making it a prominent factor leading to MDR.

In lung cancer patients, clinical studies have shown a correlation between therapeutic outcome and ABCG2 expression level (23–26). In one of these studies, the response rate to chemotherapy of patients with ABCG2-negative tumors was 44% compare to 24% in patients with ABCG2-positive tumors (27). Due to the critical role that ABCG2 plays in MDR, the search for effective ABCG2 modulators became pertinent to overcoming drug resistance. To date, several drugs have been identified as ABCG2 modulators, namely fumitremorgin C (28), Ko143 (29), gefitinib (30), and erlotinib (31).

In this study, we aimed to investigate whether M3814 can modulate ABCG2-mediated MDR in lung cancer. We hypothesized that combining M3814 with ABCG2 substrate-drugs can overcome MDR and provide a new treatment strategy for MDR cancer patients.

## Materials and Methods

### Reagents

M3814 was kindly provided by ChemieTek (Indianapolis, IN, USA). FBS, penicillin/streptomycin, DMEM, and trypsin EDTA were purchased from Corning Incorporated (Corning, NY, USA). Mitoxantrone, [^3^H] (2.5 Ci/mmol) was purchased from Moravek Biochemicals, Inc. (Brea, CA, USA). The Alexa Fluor 488 conjugated IgG secondary antibody and GAPDH monoclonal antibody (GA1R) (catalog number MA5-15738) were obtained from Thermo Fisher Scientific Inc (Rockford, IL, USA). Primary antibody against ABCG2, clone BXP-21 (catalog number MAB4146), was obtained from Millipore (Billerica, MA, USA). Anti-mouse IgG, HRP-linked antibody (catalog number 7076S) was purchased from Cell Signaling Technology Inc (Danvers, MA, USA). PBS and BSA were purchased from VWR chemicals, LLC (Solon, OH, USA). Ko143 was purchased from Enzo Life Sciences (Farmingdale, NY, USA). Paraformaldehyde, triton X-100, 4',6-diamidino-2-phenylindole (DAPI), mitoxantrone, doxorubicin, vincristine, verapamil, cisplatin, methylthiazolyldiphenyl-tetrazolium bromide (MTT), DMSO, and all other chemicals were requested from Sigma Chemical Co (St. Louis, MO, USA).

### Cell Lines and Cell Culture

The ABCG2-overexpressing subline NCI-H460/MX20 was originally established by selecting and maintaining parental NSCLC cell line NCI-H460 with mitoxantrone up to 20 nM (32). Another ABCG2-overexpressing subline A549/MX10 was originally established by selecting and maintaining parental NSCLC cell line A549 with mitoxantrone up to 10 nM (32). NCI-H460, NCI-H460/MX20, A549, and A549/MX10 were kindly provided by Drs. Susan Bates and Robert Robey (NCI, NIH, Bethesda, MD).The human embryonic kidney HEK293/pcDNA3.1 and HEK293/ABCG2 were generated by transfecting the cells with either an empty vector pcDNA3.1 or a pcDNA3.1 vector containing a full length ABCG2 gene. Transfected cells were selected with DMEM containing G418 (2 mg/mL). The ABCB1-overexpressing subline KB-C2 was established by introducing increasing concentrations of colchicine to parental cell line KB-3-1 and maintained in DMEM with 2 μg/mL colchicine (33). Both resistant KB-C2 and parental KB-3-1 cells were kindly provided by Dr. Shin-Ichi Akiyama (Kagoshima University, Kagoshima, Japan). All cells were cultured in DMEM with 10% FBS and 1% penicillin/streptomycin and maintained at 37°C incubator supplied with 5% CO_2_. The drug-selected cells were cultured in drug-free DMEM for at least 2 weeks before the experiment.

### MTT Assay

The cytotoxicity of M and other ch3814emotherapeutic agents was determined by MTT assay (34). Briefly, after cells were harvested and re-suspended in DMEM, cells were seeded evenly into 96-well plates at a density of 5,000–6,000 cells/well and incubated overnight to allow for attachment. For combination studies, an ABCG2 substrate drug and a reversal agent were added to the designated wells on the second day. After 72 h of treatment, cells were incubated with MTT. After 4 h incubation with MTT, the medium was aspirated and DMSO was used to dissolve the resulting formazan crystals. Absorbance at 570 nm was measured using the accuSkanTM GO UV/Vis Microplate Spectrophotometer (Fisher Sci., Fair Lawn, NJ, USA). Resistance-fold was calculated by dividing the IC_50_ value in resistant cells, in the presence or absence of M3814 or Ko143, by the IC_50_ value of the parental cells.

### Western Blotting Analysis

The cell lysates were collected from drug-sensitive NCI-H460 and drug-resistant NCI-H460/MX20 cells after treatment with M3814 for different time points. After protein quantitation using Pierce™ BCA Protein Assay Kit (Thermo Scientific, Rockford, IL), the protein samples were separated by PAGE then transferred onto PVDF membranes. After blocking with 5% non-fat milk, the membranes were incubated with primary antibodies against ABCG2 or GAPDH (1:1000) at 4°C overnight. Next, the blots were further incubated with HRP-linked secondary antibody (1:1000) for 2 h at room temperature. Pierce™ ECL Western blotting substrate (Thermo Scientific, Rockford, IL) was used to develop and visualize the protein bands. The results were analyzed by ImageJ software. The expression levels of ABCG2 relative to GAPDH were calculated.

### Immunocytochemistry

Cells were seeded into 24-well plates at a density of 2 × 10^4^ cells per well and incubated overnight. The cells were then treated with 1 μM of M3814 for different time points. Thereafter, the cells were fixed with 4% formaldehyde, permeabilized by 0.25% Triton X-100, and blocked with 6% BSA. Cells were then incubated with primary antibody against ABCG2 (1:1000). At the following day, primary antibody was removed, and the cells were further incubated with Alexa Fluor 488 conjugated secondary antibody (1:1000) at room temperature for 2 h. DAPI solution was added to stain the cell nuclei. Cell images were taken using a Nikon TE-2000S fluorescence microscope (Nikon Instruments Inc., Melville, NY, USA).

### Tritium-Labeled Mitoxantrone Accumulation and Efflux Assay

NCI-H460 and NCI-H460/MX20 cells were seeded at a density of 2 × 10^5^ cells per well into 24-well plates and incubated overnight to allow for attachment. Each plate was incubated with M3814 or Ko143, a positive control inhibitor of ABCG2, for 2 h at 37°C. Subsequently, cells were incubated with complete DMEM containing 10 nM of [^3^H]-mitoxantrone with or without a reversal agent for different time points. For accumulation and efflux assay in the presence of 2,4-dinitrophenol, cells were incubated in glucose-free DMEM with 1 mM 2,4-dinitrophenol for 10 min before the addition of M3814 and [^3^H]-mitoxantrone (35). Thereafter, the cells were rinsed with PBS and collected with scintillation vials. The radioactivity was read using the Packard TRICARB 1900CA liquid scintillation analyzer (Packard Instrument, Downers Grove, IL).

### Evaluation of ABCG2 ATPase Activity

The ABCG2 ATPase activity was determined using PREDEASY ATPase Kits (TEBU-BIOnv, Boechout, Belgium) with modifications as previous described (36). Briefly, different concentrations of M3814 with or without Na_3_VO${}_{4}^{-}$ were added to the ABCG2 membrane suspension. The mixtures were incubated at 37°C for 5 min and the reaction was initiated by the addition of 5 mM Mg^2+^ATP. After a 40-min incubation at 37°C, the inorganic phosphate (Pi) released was determined colorimetrically. The changes of relative light units were determined by comparing Na_3_VO${}_{4}^{-}$-treated group with the corresponding M3814-treated groups.

### M3814 Accumulation Assay

Cells were seeded at a density of 2 × 10^5^ cell per well into a 6-well-plate with a total volume of 2 mL complete DMEM. The plates were then incubated for 2 days before assay. At the day of treatment, the media was replaced by plain media (DMEM without FBS) for each well-before the drug exposure, cells were incubated with 10 mg/mL M3814 for 2 h. Thereafter, cells were washed twice with PBS followed by adding 0.5% Sodium dodecyl sulfate and acetonitrile to lyse the cells for the drug extraction. Samples were collected and centrifuged for 10 min at 14,000 rpm. The supernatant was collected and the intracellular concentration of drug was analyzed by HPLC.

### Molecular Docking

The previously reported human ABCG2 cryo-EM structure model (PDB code: 6ETI) was used for docking analysis (37). The molecular docking was performed as described (38, 39) using the Maestro v11.1 software (Schrödinger, LLC, New York, NY, USA). The best docked-conformation of M3814 and ABCG2 transporter was established through the Glide XP (extra precision) docking analysis after the ligands were prepared in the low-energy pose. The top-score results were selected and subjected to induced-fit docking with the default protocol.

### Statistical Analysis

At least 3 independent experiments were performed for each assay. Data are expressed as mean ± SD and analyzed using Graph Pad prism software 7. The data were analyzed using one-way ANOVA and statistical significance level was set as *p* < 0.05.

## Results

### M3814 Reversed ABCG2-Mediated Drug Resistance in Cancer Cells

**Figure 1:**
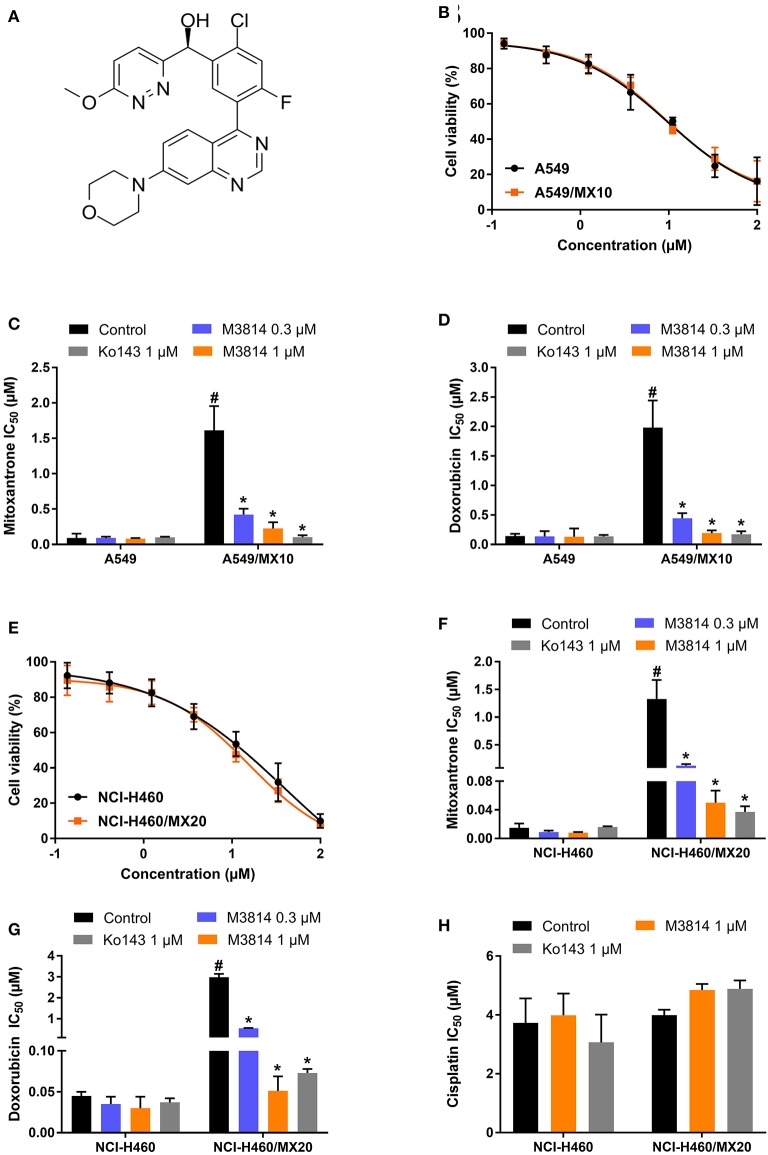
Chemical structure and the effect of M3814 on the cytotoxicity of anticancer drugs in ABCG2-overexpressing cancer cells. **(A)** Chemical structure of M3814; **(B)** Cell viability curves for A549 and A549/MX10 cells; The effect of M3814 on the cytotoxicity of mitoxantrone **(C)**, doxorubicin **(D)** in A549 and A549/MX10 cells; **(E)** Cell viability curves for NCI-H460 and NCI-H460/MX20 cells; The effect of M3814 on the cytotoxicity of mitoxantrone **(F)**, doxorubicin **(G)**, and cisplatin **(H)** in NCI-H460 and NCI-H460/MX20 cells. Data are expressed as mean ± SD from a representative of three independent experiments. *p < 0.05 vs. the control group, ^#^*p* < 0.05 vs. the control group in parental cell lines.

The chemical structure of M3814 is presented in Figure 1A. Firstly, the cytotoxicity of M3814 was determined by MTT assay. From the viability curve (Figures 1B,E), non-toxic concentrations were selected to circumvent the additive toxicity of M3814 combined with chemotherapeutic agents. Then the reversal effect was evaluated in the presence of an ABCG2 substrate drug, mitoxantrone or doxorubicin. As shown in Figures 1C-G, ABCG2-overexpressing NCI-H460/MX20 and A549/MX10 cells were highly resistant to both mitoxantrone and doxorubicin. Combining one of these substrates with M3814 or Ko143, a well-established ABCG2 inhibitor, was able to significantly sensitize the drug-resistant cells to ABCG2 substrate drugs. Furthermore, the reversal effect of M3814 at 1 μM was comparable to that of Ko143. On the other hand, M3814 did not affect the antiproliferative effect of cisplatin, a drug that is not a substrate of ABCG2, in neither drug-sensitive NCI-H460 nor drug-resistant NCI-H460/MX20 cells (Figure 1H). The cytotoxicity of cisplatin was also unaltered in drug-sensitive A549 and drug-resistant A549/MX10 cells (data not shown).

### M3814 Reversed ABCG2-Mediated Drug Resistance in Transfected Cells

**Figure 2:**
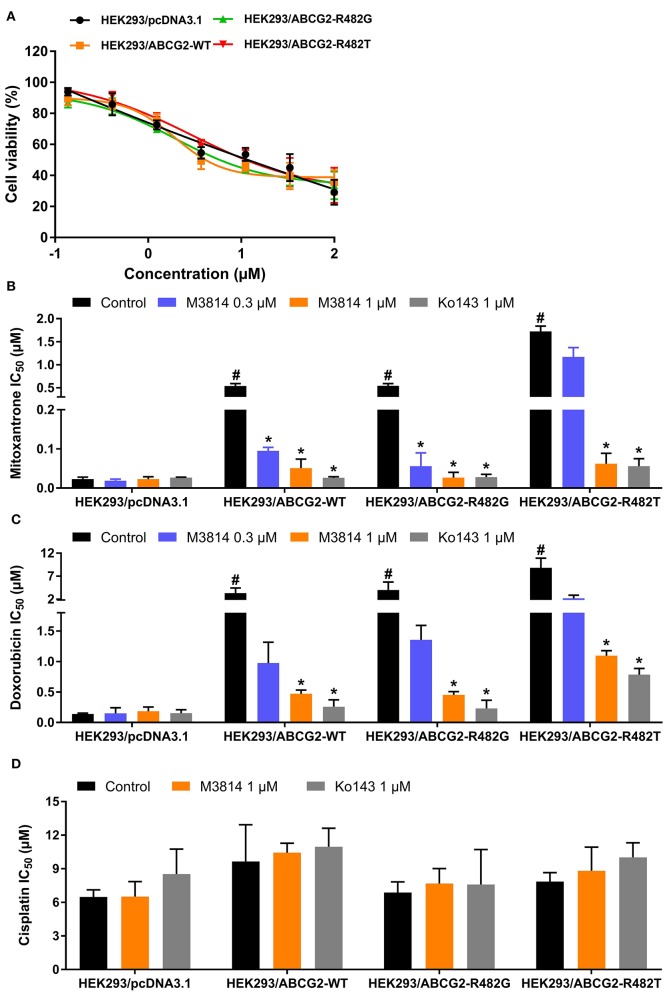
The effect of M3814 on the cytotoxicity of different anticancer drugs in ABCG2-overexpressing HEK293 transfected cells. **(A)** Cell viability curves for HEK293/pcDNA3.1, HEK293/ABCG2-WT, HEK293/ABCG2-R482G, and HEK293/ABCG2-R482T cells; The effect of M3814 on the cytotoxicity of mitoxantrone **(B)**, doxorubicin **(C)**, and cisplatin **(D)**. Data are expressed as mean ± SD from representative of three independent experiments. *p < 0.05 vs. the control group, ^#^*p* < 0.05 vs. the control group in parental cell lines.

In order to further validate the reversal effect of M3814, HEK293 transfected cells in which ABCG2 is the sole contributor to MDR were used. In short, HEK293 cells transfected with an empty vector pcDNA3.1 were regarded as the parental cells, and cells transfected with a vector containing wild-type (R482R) or mutant (R482G/R482T) ABCG2 were regarded as the drug-resistant cells. The cytotoxicity results are presented in Figure 2. Likewise, M3814 showed similar cytotoxicity in HEK293 transfected cells to cancer cells. Consistently, M3814 was able to significantly reverse drug resistance in both wild-type and mutant ABCG2 overexpressing HEK293 cells. The results support our initial finding that M3814 is a potential ABCG2 modulator.

### M3814 Did Not Affect ABCB1-Mediated MDR

**Figure 3:**
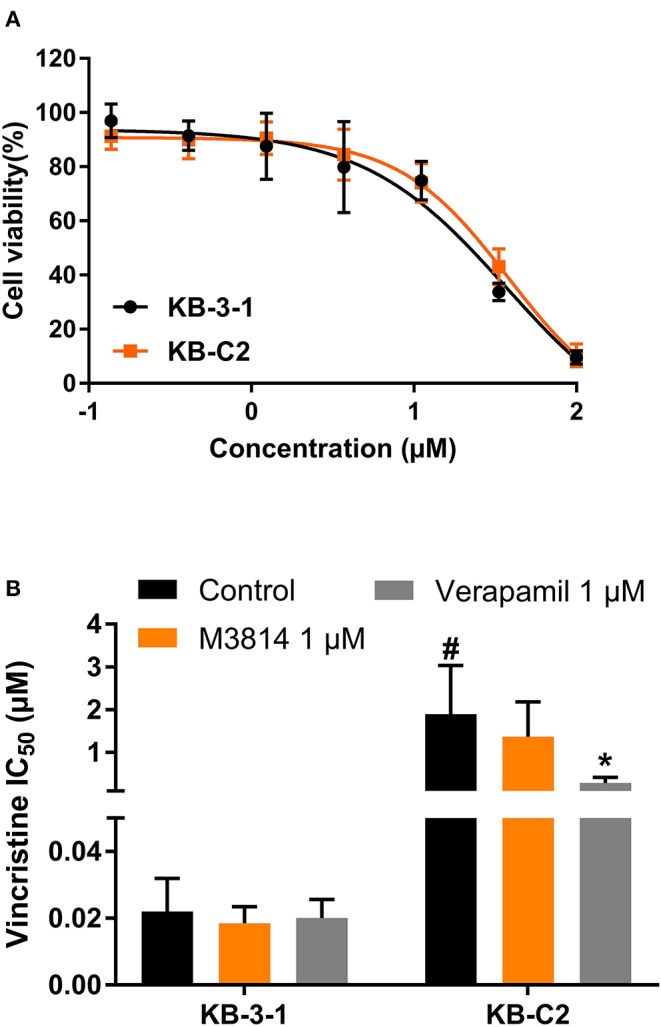
The effect of M3814 on the cytotoxicity of different anticancer drugs in ABCB1-overexpressing cancer cells. **(A)** Cell viability curves for KB-3-1 and KB-C2 cells; **(B)** The effect of M3814 on the cytotoxicity of vincristine. Data are expressed as mean ± SD from a representative of three independent experiments. *p < 0.05 vs. the control group. #*p* < 0.05 vs. the control group in parental cell lines.

To evaluate the selectivity of M3814 as an ABC drug transporter modulator, we examined whether M3814 can reverse ABCB1-mediated MDR. As shown in Figure 3A, the antiproliferative effect of M3814 in parental KB-3-1 and drug-resistant KB-C2 cells were identical and no significant toxicity was observed at 1 μM. Reversal studies showed that M3814, at 1 μM, failed to sensitize drug-resistant KB-C2 cells to vincristine, indicating that M3814 is not an effective modulator of ABCB1 (Figure 3B). Therefore, the modulatory effect of M3814 may be specific to the ABCG2 transporter.

### M3814 Increased Intracellular Accumulation and Decreased Efflux of [^3^H]-Mitoxantrone

**Figure 4:**
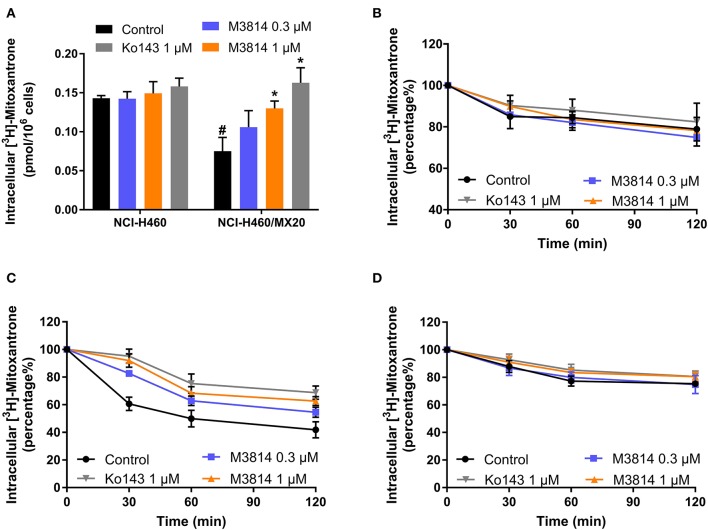
Effect of M3814 on the accumulation and efflux of [^3^H]-mitoxantrone. **(A)** The effect of M3814 on the accumulation of [^3^H]-mitoxantrone in NCI-H460 and NCI-H460/MX20 cells. **(B)** The effect of M3814 on the efflux of [^3^H]-mitoxantrone in NCI-H460 cells. **(C)** The effect of M3814 on the efflux of [^3^H]-mitoxantrone in NCI-H460/MX20 cells. **(D)** The effect of M3814 on the efflux of [^3^H]-mitoxantrone in NCI-H460/MX20 cells with 2,4-dinitrophenol pretreatment. Ko143 at 1 μM was used as a positive control inhibitor of ABCG2. Data are expressed as mean ± SD from a representative of three independent experiments. *p < 0.05 vs. the control group, ^#^*p* < 0.05 vs. the control group in parental cell lines.

M3814 showed the ability to reverse ABCG2-mediated MDR, and we therefore, sought to further investigate its modulatory mechanisms. [^3^H]-mitoxantrone accumulation and efflux assays were performed to measure the intracellular concentration of mitoxantrone. As shown in Figure 4A, the intracellular concentration of [^3^H]-mitoxantrone was lower in drug-resistant NCI-H460/MX20 cells than in drug-sensitive NCI-H460 cells without the presence of a reversal agent. The addition of M3814 or Ko143 increased the accumulation of [^3^H]-mitoxantrone in drug-resistant NCI-H460/MX20 cells without affecting accumulation in drug-sensitive NCI-H460 cells. The results provided evidence that M3814 can increase the accumulation of [^3^H]-mitoxantrone in ABCG2-overexpressing cells. Since there are multiple factors that can result in increased mitoxantrone accumulation, we first explored whether M3814 can inhibit the efflux function of ABCG2. As shown in Figures 4B,C, without altering the efflux process in drug-sensitive cells, M3814 was able to hinder the efflux of [^3^H]-mitoxantrone in drug-resistant cells. 2,4-dinitrophenol is an uncoupling agent that blocks the phosphorylation of ADP to ATP. We measured the efflux process of [^3^H]-mitoxantrone in NCI-H460/MX20 cells in glucose-free DMEM in the presence of 2,4-dinitrophenol. As presented in Figure 4D, the efflux of [^3^H]-mitoxantrone was decreased in NCI-H460/MX20 cells by 2,4-dinitrophenol while the addition of Ko143 or M3814 had no significant effect. Taken together, the results suggested M3814 can inhibit the efflux activity of the ABCG2 transporter, which leads to the increased intracellular concentration of mitoxantrone.

### M3814 Stimulated ABCG2 ATPase Activity

**Figure 5:**
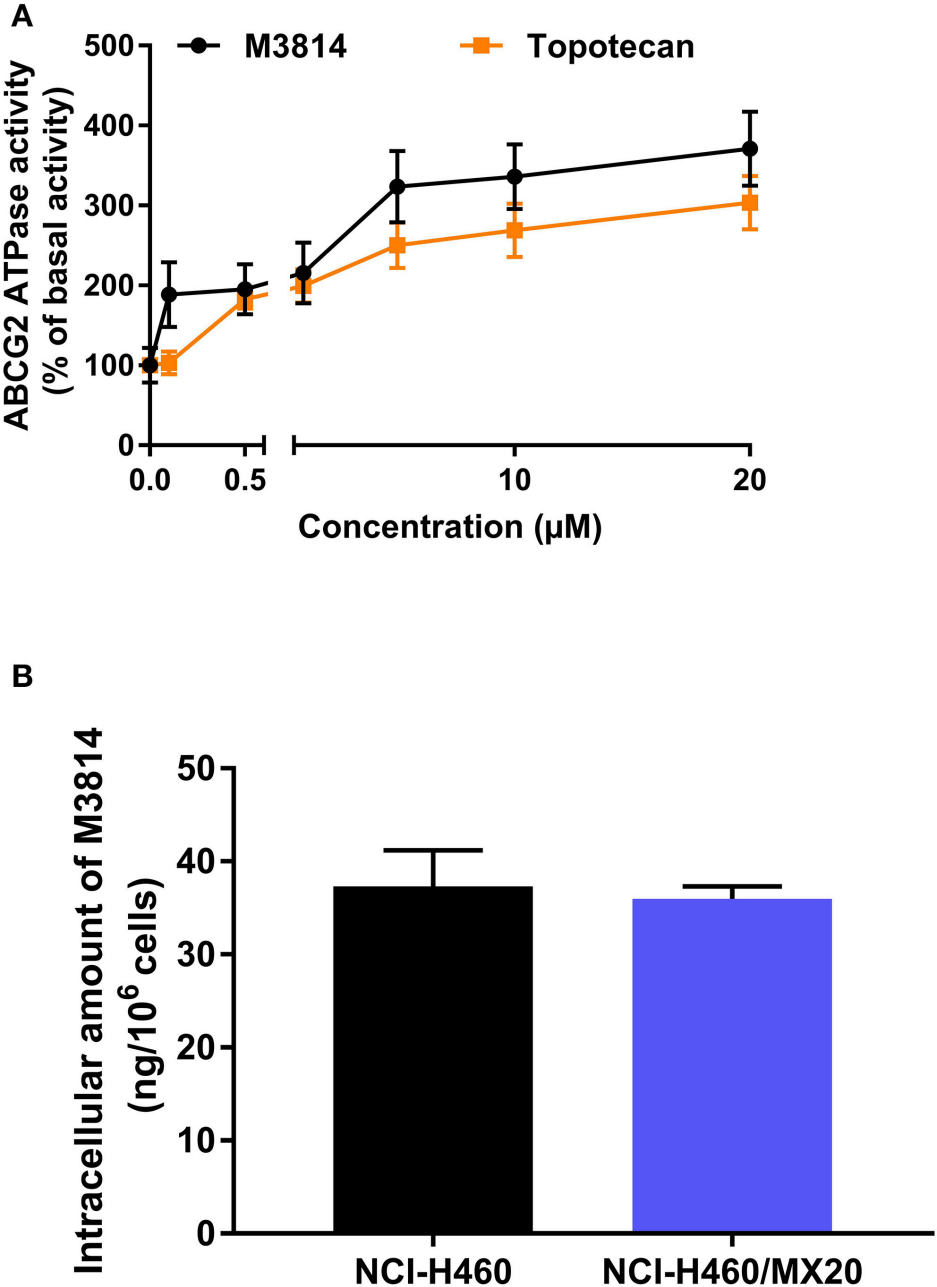
M3814 stimulates ABCG2 ATPase activity without being transported out of the resistant cells **(A)** The effect of M3814 and topotecan on the ATPase activity of ABCG2 transporter. **(B)** The intracellular accumulation of M3814 in NCI-H460 and NCI-H460/MX20. Data are expressed as mean ± SD from three independent experiments.

The [^3^H]-mitoxantrone accumulation and efflux assay suggested that M3814 can interact with the ABCG2 transporter. We postulated that M3814 may be a direct ABCG2 inhibitor or an ABCG2 substrate that can inhibit or stimulate the ATPase function, respectively. Therefore, the ABCG2 ATPase assay was conducted to evaluate the role of M3814. As presented in Figure 5A, M3814 showed a concentration-dependent stimulation of ATPase activity at 0–20 μM. The stimulatory effect reached 50% maximal effect at 1.64 μM with a maximum stimulation of 371.02% of the basal activity. In this study, topotecan was used as a positive substrate control that can stimulate the activity of ABCG2 ATPase. Combined with the results of the accumulation and efflux assay, M3814 may be a transported substrate and competitively inhibit the transportation of other ABCG2 substrates.

### The Intracellular Accumulation of M3814 Was Consistent in Parental and Drug-Resistant Cells

Since M3814 can stimulate ABCG2 ATPase activity, we examined whether M3814 can be pump out of the cells by ABCG2. By performing HPLC analysis, it is found that the intracellular accumulation of M3814 showed no significant different in parental NCI-H460 and ABCG2-overexpressing NCI-H460/MX20 cells (Figure 5B). These results suggest that M3814 can bind to ABCG2 transporter without being pump out of the cells.

### M3814 Did Not Affect ABCG2 Protein Level or Cell Surface Localization

**Figure 6:**
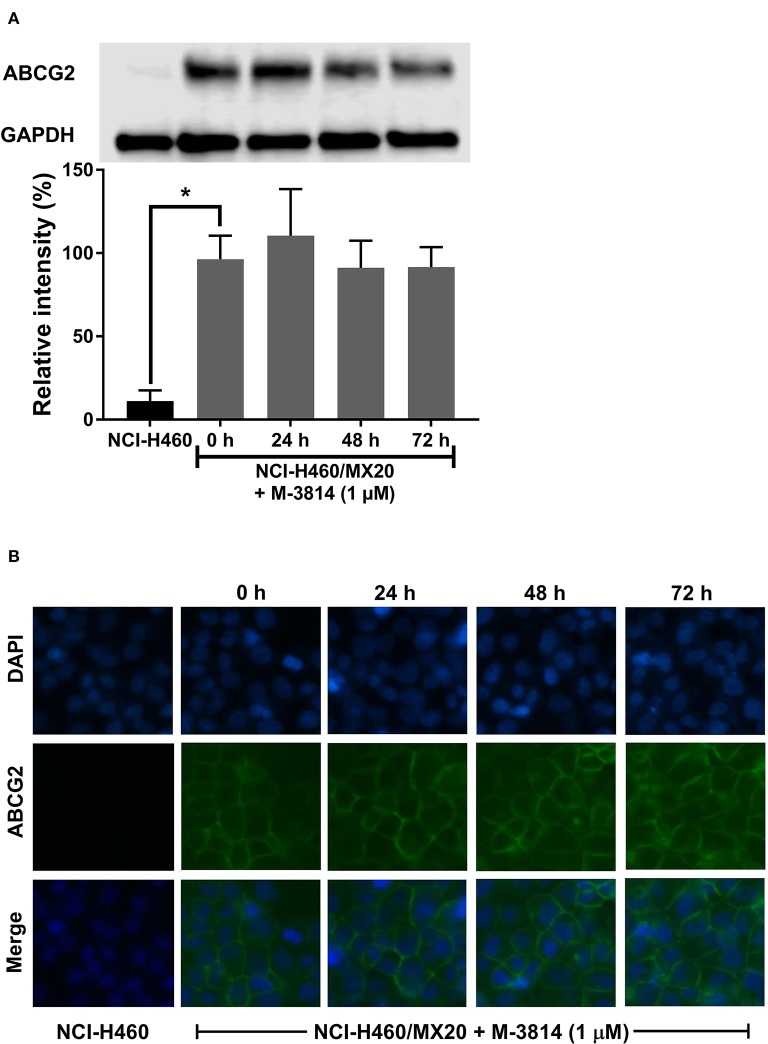
M3814 did not affect the ABCG2 protein expression or cell surface localization. **(A)** The effect of M3814 on the expression level of ABCG2 in NCI-H460 and NCI-H460/MX20 cells. **(B)** Cell surface localization of ABCG2 expression in NCI-H460/MX20 cells incubated with 1 μM of M3814 for up to 72 h. Data are expressed as mean ± SD from three independent experiments. *p < 0.05 vs. the control group.

Since a reversal agent may exert its effect through multiple mechanisms, we investigated whether M3814 can alter ABCG2 protein expression or cell surface localization. As shown in Figure 6A, M3814 did not alter the expression level of ABCG2. Furthermore, the immunofluorescent assay (Figure 6B) clearly showed that ABCG2 continued to be localized at cell surface after 72 h of M3814 treatment. These results indicated that the modulatory effect of M3814 may be due solely to the inhibition of substrate efflux. Similarly, ABCG2 expression level and cell surface localization were not changed in A549/MX10 cells after M3814 treatment (data not shown).

### Molecular Docking Analysis of M3814 With the Human ABCG2 Homology Model

**Figure 7:**
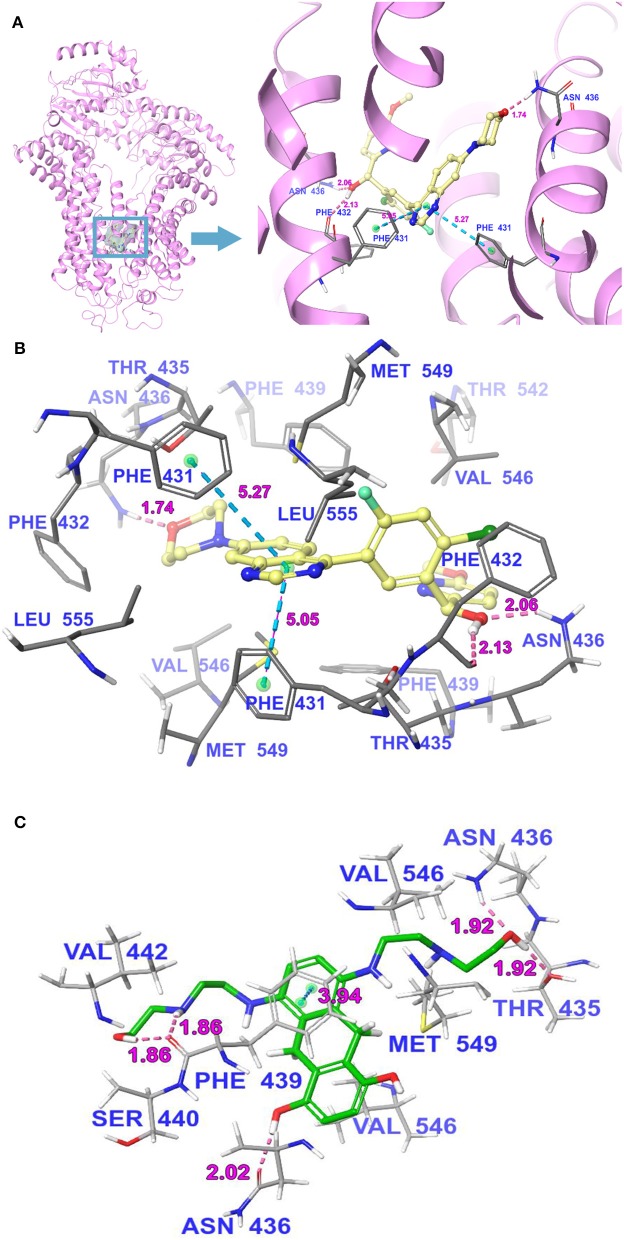
The molecular binding mode of M3814 to the human ABCG2 model as predicted by induced-fit docking. **(A)** The superimposition of the best-scored pose of M3814 within the binding cavity of ABCG2. Ligand is depicted as a ball-and-stick model with a transparent gray surface and the ABCG2 structure as a ribbon diagram in faded pink. The detailed depiction of M3814 and nearby residues inside the ABCG2 binding cavity are depicted with the atoms colored as follows: carbon, faded yellow; hydrogen, white; nitrogen, blue; oxygen, red; fluorine, light green; chlorine, dark green; and important amino acid residues are described (sticks model) with the same color scheme as above for all atoms but carbon atoms in gray. The values of the relevant distances are indicated in Å. **(B)** The docked conformation of M3814 (ball-and-stick model) is shown within the ABCG2 drug-binding cavity, and specific amino acids residues within 3 Å are indicated as a sticks model, with the same color scheme as **(A)**. Dotted pink lines represent hydrogen-bonding interactions, while dotted light blue lines represent π-π stacking interactions. The values of the correlation distances are indicated in Å. **(C)** The docked conformation of mitoxantrone (ball and stick model) is shown within the ABCG2 drug-binding cavity with the atoms and important amino acid residues are described (sticks model) with the same color scheme as above for all atoms but carbon atoms in green. Dotted pink lines represent hydrogen-bonding interactions, while dotted light blue lines represent π-π stacking interactions. The values of the correlation distances are indicated in Å.

The interactions between M3814 and human ABCG2 model were stimulated by the induced-fit docking analysis. The Glide gscore of the best docked pose of M3814-ABCG2 was −14.035 kcal/mol, which indicated that M3814 has a good affinity to the drug-binding pocket of ABCG2. Figure 7A depicted the general docking pose of M3814, which was predicted in the drug-binding cavity of human ABCG2 model with the detailed interactions between M3814 and some specific residues of the protein model. As shown in Figure 7B, the best-scored pose of M3814 was mainly stable in the ABCG2 transmembrane domain, demonstrating hydrophobic interactions with specific residues including Leu555, Phe431, Phe432, and Phe439 in protein chain A. Other hydrophobic interactions include Leu555, Val546, Met549, Phe431, Phe432, and Phe439 in protein chain B. Furthermore, Figure 7B also depict that a hydrogen bond was formed between the ether group in morpholine of M3814 and Asn436 of ABCG2 chain A. In addition, M3814 formed two hydrogen bonds with protein chain B: one between Phe432 and the methanol group of M3814, another one between Asn436 and the same methanol group. Two π-π stacking interactions were formed between the quinazoline ring of M3814 and Phe431 of both ABCG2 chain A and chain B, respectively. The molecular docking analysis of mitoxantrone with ABCG2 model was shown in Figure 7C. M3814 only have two overlapping binding residues, indicating M3814 may not bind to the same substrate-binding site as mitoxantrone.

## Discussion

As one of the more well-known members of the ABC transporter family, ABCG2 can be both beneficial and deleterious. By eliminating xenobiotics from cells, ABCG2 acts as a gatekeeper in normal tissue but as an MDR mediator in many tumors (40). ABCG2 overexpression can confer cancer cells resistant to a wide range of chemotherapeutic agents such as irinotecan, doxorubicin, and mitoxantrone. Due to its pivotal role, tremendous effort has been devoted to investigating ABCG2 inhibitors. Even though the clinical effect of these ABCG2 inhibitors remains inconclusive, an appropriate modulation of ABCG2 activity may strengthen the efficacy of substrate-drugs by overcoming MDR and improving their pharmacokinetics (41–43). Recent studies suggest that the combination of several TKIs with substrate-drugs can achieve desired effect and reverse drug resistance (44–46).

Lung cancer accounts for a large proportion of cancer incidences and mortalities. About 85% of the cases are characterized as NSCLC. Clinical data have shown that ABCG2 overexpression may attenuate the response of NSCLC patients to anticancer drug. M3814 is developed as a potent and selective DNA-PK inhibitor. It is now under several clinical trials for advanced solid tumors and CLL, as well as in combination treatment with radio-chemotherapy. In this study, the ABCG2 modulatory effect of M3814 was evaluated in two ABCG2-overexpressing NSCLC cell lines, NCI-H460/MX20 and A549/MX10. We report that M3814 can effectively modulate the function of ABCG2 and reverse MDR in combination treatment.

The cytotoxicity of M3814 was examined in both parental and ABCG2-overexpressing cell lines to select the appropriate concentrations for reversal studies. The results showed that no significant toxicity was observed up to 1 μM in both sets of NSCLC cell lines. Therefore, the reversal experiments were carried out using 0.3 and 1 μM of M3814 to evaluate its reversal effect. The data showed that M3814 can significantly sensitize the drug-resistant cells to ABCG2 substrates (mitoxantrone and doxorubicin) but this phenomenon was not observed in the drug-sensitive parental cells, suggesting that the effect may be due to ABCG2 efflux inhibition. Furthermore, it was documented that a mutation at the position 482 of the ABCG2 protein can result in a distinct substrate-binding and efflux profile (47, 48). By conducting reversal studies in gene-transfected HEK293 cells, we confirmed that M3814 can reverse both wild-type and mutant ABCG2-mediated MDR in cellular models. Comparing with other established ABCG2 modulators, the effective concentration of M3814 is lower than ulixertinib (49), selonsertib (50), ribociclib (51), PD1530353 (52), and comparable to Ko143, olmutinib (53), SIS3 (54). M3814 did not alter the toxicity of cisplatin, a non-substrate drug of ABCG2, nor did it reverse ABCB1-mediated MDR. Therefore, M3814 may be a modulator specific to ABCG2. Although we uncovered that M3814 may not be a reversal agent for ABCB1-mediated MDR, future studies are needed to assess the interaction of M3814 with other MDR-associated proteins such as ABCC1 and ABCC10 (55, 56).

Further studies were conducted to gain insight into the reversal mechanisms. [^3^H]-mitoxantrone accumulation and efflux assays were conducted. By introducing the tritium-labeled mitoxantrone, it allowed for a direct measurement of intracellular concentration of mitoxantrone to assess the efflux process of ABCG2. From the results of the accumulation assay, we found that M3814 can increase the accumulation of mitoxantrone only in ABCG2-overexpressing cells in a concentration-dependent manner, indicating that M3814 can interact with an ABCG2 transporter to interfere with its efflux function. Consistently, in the [^3^H]-mitoxantrone efflux assay, M3814 was able to hinder the efflux process in ABCG2-overexpressing cells and the effect was comparable with the well-established ABCG2 inhibitor Ko143. To determine if the enhanced mitoxantrone accumulation is due to the decreased active efflux or increased drug uptake by the cells, we introduced 2,4-dinitrophenol which can prevent the synthesis of ATP and thereby can deplete the intracellular ATP. As ABCG2 efflux function requires energy from ATP hydrolysis, pretreatment with 2,4-dinitrophenol can significantly decrease the drug efflux process. Our results showed that, in the presence of 2,4-dinitrophenol, ABCG2-overexpressing NCI-H460/MX20 cells showed significant decreased efflux of mitoxantrone, suggesting the efflux function of ABCG2 is impaired. Furthermore, adding reversal agents M3814 or Ko143 did not contribute to the accumulation of mitoxantrone after cells were pretreated with 2,4-dinitrophenol. These results suggest that M3814 can hinder the ABCG2 efflux function without facilitating mitoxantrone uptake by the cells. Therefore, the modulatory effect of M3814 may be in part due to the inhibition of ABCG2 efflux function, which leads to increased substrate accumulation and enhanced cytotoxic effect.

Since we demonstrated that M3814 can attenuate the efflux activity of ABCG2, it may act by inhibiting the ATPase, hindering ABCG2 from utilizing the energy from ATP hydrolysis. Another explanation, according to several previous studies (49, 50, 57), is that the modulator may act as a substrate of ABCG2 and compete with other substrates for the transporter. To determine the mechanism of M3814 as an ABCG2 modulator, ABCG2 ATPase activity was measured in the presence of M3814. ABCG2 is characterized as a “half-transporter” with a transmembrane domain to which a substrate binds and a nucleotide-binding domain where ATP binds and hydrolyzes. If the drug is an ATPase inhibitor, a trend toward decreased ATPase activity would be observed (36). In contrast, a substrate can bind to the substrate-binding pocket and stimulate the ATPase to provide energy for drug efflux. The results indicated that M3814 can stimulate the activity of ABCG2 ATPase in a concentration-dependent manner, confirming its role as a transported substrate of ABCG2. Some ABCG2 substrate inhibitors can act as competitive inhibitors that bind to a distinct substrate-binding site and inhibit the efflux of a particular class of substrates (58). Another possibility is that some substrate inhibitors can interact with ABCG2 on sites other than the substrate-binding sites and cause conformational changes in the binding pocket which allosterically affect the transportation of some substrates (59). It should be noted that, although several ABCG2 inhibitors were identified as substrates through ATPase assay, overexpression of ABCG2 does not necessarily confer drug resistance to these inhibitors (49, 54, 60–62). Hitherto, the detailed mechanism of this inhibitory effect remained inconclusive and desire further exploration. To explore the possible mechanism of the reversal effect achieved by M3814, we performed HPLC to detect the intracellular accumulation of M3814 in parental and drug-resistant cells. The results showed that ABCG2-overexpressing cells did not 14. Sinc necessarily efflux M38e M3814 can stimulate the activity of ABCG2 ATPase, we hypothesized that M3814 can bind to the drug-binding pocket of ABCG2, causing the protein conformational changes and therefore inhibit the efflux of other substrates. Further studies are needed to verify this possible mechanism of action.

Subsequently, we examined two additional possible mechanisms: alteration of ABCG2 protein expression or cell surface localization. Some reversal agents can downregulate the ABCG2 expression, leading to decreased drug resistance (52). The reversal effect can be achieved by translocating the transporter into cytoplasm, which also decreases the number of efflux pumps on the cell membrane. By performing a Western blot and immunofluorescent assay, we confirmed that M3814 did not affect the expression level of ABCG2, and the transporter remained on the cell membrane after M3814 treatment. Hence, we inferred that M3814 can inhibit the efflux function of ABCG2. Further studies are needed to explore other potential mechanisms.

Molecular docking has been extensively used in the field of structural molecular biology, and helps to predict the predominant binding interaction between a ligand and a protein. The cryo-EM structures of ABCG2 bound with an ABCG2 inhibitor Ko143 (37) were used as the basis for investigating the interaction between ABCG2 modulators and the transporter. M3814 obtained a high docking score of−14.035 kcal/mol compared to several other ABCG2 modulators such as ulixertinib (−11.501 kcal/mol) (49), PD153035 (−7.015 kcal/mol) (52), and TMP195 (−12.7 kcal/mol) (63), suggesting that M3814 has potent binding affinity with the drug-binding cavity of ABCG2. Of note, the molecular docking is not meant to be accurate affinity predictor, thereby the bound conformation may not represent the actual binding situation and is therefore used as a reference for this study (64).

In conclusion, our study highlights M3814 as a modulator of ABCG2 function. The combination of M3814 and ABCG2 substrate drugs may allow additional benefit for cancer patients with high ABCG2 expression.

## Data Availability Statement

The datasets generated for this study are available on request to the corresponding author.

## Author Contributions

Z-XW, Z-SC, CZ, and LLin: Conceptualization. Z-XW, ZP, YY, Q-XT, J-QW, Z-NL, Y-GF, KP, and LLiu: methodology. Z-XW: writing - original draft preparation. Z-XW, YY, LLin, CZ, and Z-SC: writing - review and editing. Z-SC and CZ: supervision.

## Conflict of Interest

The authors declare that the research was conducted in the absence of any commercial or financial relationships that could be construed as a potential conflict of interest.
